# Hyperacusis in Autism Spectrum Disorders

**DOI:** 10.3390/audiolres11040049

**Published:** 2021-10-14

**Authors:** Ali A. Danesh, Stephanie Howery, Hashir Aazh, Wafaa Kaf, Adrien A. Eshraghi

**Affiliations:** 1 Department of Communication Sciences and Disorders, Florida Atlantic University, Boca Raton, FL 33431, USA; stephaniehoweryslp@gmail.com; 2 Department of Integrated Medical Sciences, Schmidt College of Medicine, Florida Atlantic University, Boca Raton, FL 33431, USA; 3 Department of Audiology, Tinnitus and Hyperacusis Specialty Clinic, Royal Surrey Hospital, Guildford GU2 7XX, UK; hashir.aazh@nhs.net; 4 Communication Sciences and Disorders Department, Missouri State University, Springfield, MO 65897, USA; WafaaKaf@MissouriState.edu; 5 Department of Otolaryngology and Neurological Surgery, Miller School of Medicine, University of Miami, Miami, FL 33146, USA; aeshraghi@med.miami.edu

**Keywords:** audiology, auditory system, decreased sound tolerance disorder, hyperacusis, misophonia, noise sensitivity, tinnitus, autism spectrum disorder

## Abstract

Hyperacusis is highly prevalent in the autism spectrum disorder (ASD) population. This auditory hypersensitivity can trigger pragmatically atypical reactions that may impact social and academic domains. Objective: The aim of this report is to describe the relationship between decreased sound tolerance disorders and the ASD population. Topics covered: The main topics discussed include (1) assessment and prevalence of hyperacusis in ASD; (2) etiology of hyperacusis in ASD; (3) treatment of hyperacusis in ASD. Conclusions: Knowledge of the assessment and treatment of decreased sound tolerance disorders within the ASD population is growing and changing.

## 1. Introduction

Decreased Sound Tolerance Disorders (DSTD) are routinely observed in autism spectrum disorder (ASD). The most common types of DSTD are hyperacusis and misophonia. Hyperacusis is a class of decreased sound tolerance disorders in which a negative or incongruous reaction is triggered from exposure to sounds that are not described as threatening or uncomfortable by a neurotypical individual [1,2,3,4]. These reactions are in response to general sounds, rather than specific sounds (such as chewing and sniffling), as would be the case with misophonia [5]. Hyperacusis can affect an individual at various degrees depending on the severity. It can impact one’s emotional wellbeing, sleep, concentration, and can cause anxiety [6]. The precise cause of hyperacusis still remains unknown. Theories include the idea that hyperacusis is the result of increased neural synchrony and reorganization of the tonotopic structure of the auditory cortex as well as the possibility that neurons that would typically respond to loud sounds start to respond to lower intensity sounds [7]. Utilizing Magnetic Resonance Imaging (MRI), scientists have seen elevated auditory activity in the auditory midbrain, thalamus and cortex, as well as enlarged subcortical and cortical responses to sound in subjects with hyperacusis [8,9]. Other theories propose the role of central gain enhancement in hyperacusis and the possibility of hyperacusis to be an indication of problems with the limbic system or auditory pathway [10,11,12].

## 2. Hyperacusis: Assessment and Prevalence

### 2.1. Assessment of Hyperacusis 

Assessment of hyperacusis typically will involve extensive case history taking, pure tone audiometry, measurement of uncomfortable loudness levels (ULLs), and self-report questionnaires such as the hyperacusis questionnaire (HQ) [2,7,13]. However, due to limitations in obtaining accurate levels of loudness discomfort or sometimes hearing thresholds, particularly in severe cases of ASD, clinicians rely on behavioral observation strategies and case history. Generally, ULL provides the level above which tones become uncomfortably loud for a patient [7]. In patients with hyperacusis, ULLs will typically be lower than the average person with normal hearing and without hyperacusis. The average ULL for patients with normal hearing and without hyperacusis is around 100 dB hearing level (HL), and reports of ULLs for patients with hyperacusis have been reported to be around 60 to 85 dB HL [2,7]. 

Based on results obtained with adults, Aazh and Moore [14] proposed diagnostic criteria for hyperacusis based on the average ULL across 0.25, 0.5, 1, 2, 4 and 8 kHz for the ear with the lower average ULL, which is denoted ULLmin. They suggested that a value of ULLmin equal to or below 77 dB HL should be taken as indicating the presence of hyperacusis [14]. With this ULLmin criterion, 95% of adult patients diagnosed as having hyperacusis were found also to meet the criterion of a cut-off score on the Hyperacusis Questionnaire (HQ) [13] of 22 or more [14]. Interestingly, among children and adolescents seeking help for tinnitus and/or hyperacusis from an audiology clinic, the mean value of ULLmin was 64 dB HL (SD = 15, n = 34). [14]

### 2.2. Prevalence of Hyperacusis and Concomitant Diagnoses

A number of studies have analyzed the prevalence of hyperacusis in the general population [13,14]. These findings have ranged from reports of 3.2% up to 17.1% [13,14]. It has been found that hyperacusis often accompanies other medical conditions [14,15]. These diagnoses include a high number of psychiatric conditions such as post-traumatic stress disorder (PTSD), depression, and exhaustion syndrome as well as migraines, tinnitus, hearing loss, attention deficit hyperactivity disorder (ADHD), and autism spectrum disorder (ASD). In a retrospective study analyzing case notes of 61 children with hyperacusis, it was found that 28 of the children, or 46% of the sample, had a concomitant neurodevelopmental condition, with the most common diagnosis being autism spectrum disorder [15]. This is not surprising, as it is known that sound hypersensitivity is a common component of ASD. 

A pilot study in 1995 reported a 40% prevalence of hyperacusis in the ASD population [16]. More recently, Demopoulos and Lewine analyzed the audiometric profiles of 60 autistic children and adolescents ages 5 through 18 [17]. Comprehensive assessment revealed 37% of the participants to have sound sensitivity in at least one ear [17]. Rosenhall et al., 1999, studied the auditory characteristics such as hearing loss and hyperacusis of 199 children and adolescents with ASD and reported 18% prevalence for hyperacusis in their study sample [18]. Danesh and colleagues conducted a study to evaluate the presence of tinnitus and hyperacusis in individuals between the ages of 4 and 42 diagnosed with Asperger’s Syndrome [19]. It should be noted that Asperger’s Syndrome is a diagnosis that is no longer being used and has since become part of the ASD diagnosis [20]. Danesh, et al study used a home developed case history survey as well as the Tinnitus Reaction Questionnaire [21], Tinnitus Handicap Inventory [22], and Hyperacusis Questionnaire [14] to survey 55 participants. These questionnaires found that the ASD group had a much higher prevalence of both hyperacusis and tinnitus than the general population. Within this study group of ASD participants, 69% reported hyperacusis with an average Hyperacusis Questionnaire score of 20.7 [19]. In a recent metanalysis of hyperacusis in individuals with ASD, the researchers concluded that hyperacusis has a high prevalence across the life span of this population [20]. 

## 3. Hyperacusis and ASD

### 3.1. Autism Spectrum Disorder and Hyperacusis

Autism spectrum disorder (ASD) is a complex neurological and developmental condition that is characterized by a number of differences in the sensory, behavioral, language, and social domains [23,24]. ASD is referred to as a spectrum disorder because presentations of this diagnosis vary by individual. Most commonly discussed presentations include language delays, social/pragmatic differences, rigid and restricted interests and behaviors [24]. More recently, differences in attention and perceptual and sensory processing have been found to be central components to ASD as more research has found the role these differences play in communication [25]. Research on sensory profiles of autistic individuals has suggested increased sensory discrimination skills as well as increased distractibility [26]. Theories reviewed by Remington and Fairnie include the idea that autistic individuals have an increased perceptual capacity allowing them to process an increased amount of cognitive information as compared to neurotypical peers [27]. This includes visual information as well as auditory stimuli. 

It is important to note the semantic preferences in the autism community have changed over the years as the neurodiversity movement has become increasingly widespread. Research demonstrates that many members of the autistic community most commonly prefer use of identity first language (autistic person), whereas allies and professionals working were more likely to use person first language (person with autism) [28]. It is important for professionals to understand and acknowledge the preferences of autistic individuals in order to help to facilitate a positive and inclusive environment. 

Across a variety of sensory domains including auditory processing, autistic individuals may demonstrate hyper-reactivity or hypo-reactivity. It has been reported that hypersensitivity is perceived to be more of a problem impacting daily life than hyporeactivity [29]. Auditory processing differences in autistic individuals have been noted regarding sensory perception and processing of auditory stimuli. Perceptual differences have been found including superior perception of pitch, superior identification of musical notes, and superior local processing of auditory stimuli as compared to neurotypical adults [30,31]. Autistic individuals have been found to have intact global processing of auditory stimuli and reduced global interference, meaning they best process auditory stimuli at the musical note level as compared to processing a melody. In addition to enhanced abilities and differing levels of processing, autistic individuals also have been found to have difficulties with regard to auditory processing. Recent research by Vlaskamp and colleagues in 2017 has noted that autistic children demonstrate reduced mismatch negativity, suggesting that autistic children are less able to automatically encode deviant sounds [30]. In fact, auditory hypersensitivity is often a key indicator of ASD [29,31]. Hypersensitivity to auditory stimuli is exacerbated in the ASD population, resulting in sensory-based reactions such as covering one’s ears, crying, or running away [31,32,33]. Such reactions may be perceived as pragmatically atypical may adversely impact social and academic function. A summary of the prevalence of hyperacusis in ASD population from four studies is shown in Table 1. A quick glance on Table 1 shows that these percentages are more than double the prevalence of the general population.

### 3.2. Correlates of Hyperacusis in the ASD Population

A number of proposed causes of hyperacusis have been introduced, but it is important to consider that correlates of hyperacusis may differ across individual cases of ASD and hyperacusis given the extreme level of variability in the makeup of the brain depending on the level of severity of the ASD. Smith, Storti, Lukose, and Kulesza [34] reported imaging studies that demonstrated cerebellar and brainstem hypoplasia in the ASD population compared to age matched people with neurotypical development, including hypoplasia of the facial nucleus and superior olivary complex. Another interesting study highlighting anatomical causes of hyperacusis in the ASD population noted that 29% of autistic people and hyperacusis were found to have superior semicircular canal dehiscence as demonstrated by computerized tomography imaging [35]. Due to this significant percentage of superior canal dehiscence within this specific population, this study was then elaborated to find that vestibular evoked myogenic potential (VEMP) demonstrated diagnostic ability to differentiate between hyperacusis due to superior canal dehiscence and dehiscence due to bone immaturity in autistic children [36]. A 2013 study found a delayed response of stapedial acoustic reflex in the ASD group, asserting that autistic patients can be identified using this measure [37]. Another recent study assessed the correlation of loudness tolerance with the stapedial reflex threshold with contralateral suppression of distortion product otoacoustic emissions (DPOAEs), as contralateral suppression of DPOAEs is typically increased in patients with hyperacusis [38]. Results indicated that stapedial reflex was lower in the ASD group and was significantly correlated with loudness tolerance in both the ASD and control groups. This is supported by previous findings by Danesh and Kaf [39], in which DPOAEs were found to have lower amplitudes in absence of noise in the ASD group, and with contralateral noise, the suppression effect was weaker in the ASD group indicating both cochlear and efferent system lesion. Kaf and Danesh [40] also studied contralateral suppression of DPOAEs to broad band noise in 18 autistic children with the primary diagnosis of Asperger’s syndrome and 18 control group participants. Results showed no significant differences between groups for both DPOAE response signal to noise ratio with and without contralateral noise. The lack of significant differences may be because the study group was on the high-functioning end of the autism spectrum (e.g., Asperger). Kaf and Danesh [40] also suggested that the generation of hypersensitivity to sounds in high-functioning autistic children may be due to abnormal neural connections at proximal structures to the medical olivary complex, such as the temporal lobe, limbic system and autonomic nervous system. Several neuroimaging studies have shown abnormal connectivity in the brain of children with ASD such as enlarged brain volume, 10% enlargement of the white matter of the temporal lobe and corpus callosum and decreased neural cell size in ASD [41,42]. These brain abnormalities are age-specific, with faster growth in childhood [43] as well as gender specific [44]. Thus, differences in the study participants’ ages and gender may explain lack of significant findings or contradictory findings in autistic children. 

Other causes of hyperacusis within the ASD population have been attributed to reduced strength of the efferent pathway of the auditory system. Knowing that medial olivocochlear (MOC) reflex is stronger than average in neurotypical adults with hyperacusis, Wilson, Sadler, Hancock, Guinan, and Lichtenhan [45] assessed the MOC efferent reflex in autistic children using transient evoked OAE (TEOAE). Results indicated that the group of autistic participants with severe hyperacusis had MOC reflexes that were twice as strong as groups of neurotypical participants and groups of autistic participants without severe hyperacusis. Research by Ida-Eto, Hara, Ohkawara, and Narita suggested that auditory hypersensitivity in ASD may be linked to impairment of inhibitory processing of the auditory system [46]. This study found decreased expression of protein markers for inhibitory neurons (i.e., immunoreactivity) of the superior olivary complex secondary to decreased size of the medial nucleus of the trapezoid body; however, it is important to note that this was found using ASD model rats with prenatal exposure to thalidomide as opposed to transgenic animal models or autistic human subjects.

In addition to possible anatomical and physiological causes of hyperacusis, outside factors possibly contributing to hyperacusis in the autism population have been found in the literature, including a recent case study of an autistic 11 year-old boy, whose hyperacusis worsened when he was taking risperidone to treat hyperactivity and behavioral problems [47]. However, a contrasting case study noted an autistic five and a half year-old girl whose hyperacusis, according to parent report, alleviated after taking the same medication [48]. Knowing the heightening effects hyperacusis has on existing sensory-based responses in ASD, more research is needed on the possible effects of supplements or medications and the role they play in hyperacusis of autistic people. 

Genetics may also play a role in hyperacusis within the ASD population. Mertcati and colleagues [49] affirmed that contactin genes CNTN5 (contactin 5 is a protein coding gene) and CNTN6 (contactin 6 is also a protein coding gene) for neuronal cell adhesion molecules which promote neurite outgrowth in the sensory-motor pathways have been reported in autistic people. Reports included an autistic girl who had 5 copies of the CNTN5 gene, as well as multiple cases of deletion or mutation of the CNTN6 gene in other autistic individuals. Clinical investigation of patients carrying these CNTN5 and CNTN6 variants demonstrated the presence of hypersensitivity to sounds, and it was found that they had changes in wave latency of their ABR within the auditory pathway [43]. 

As previously noted, Remington and Fairnie directly assessed auditory capacity in autistic individuals [27]. They used behavioral experiments to examine the auditory processing profile of autistic individuals, with findings suggesting that autistic individuals have an increased auditory perceptual capacity compared to neurotypical individuals, which may result in sensory overload. This suggests that increased processing capacity may be the reason why autistic individuals are increasingly predisposed to hyperacusis. The authors proposed the reframing of perceptual processing of autistic individuals in terms of increased capacity as opposed to a deficit in filtering or processing sounds, in order to best develop interventions to alleviate distress in response to sensory stimuli including hyperacusis [27]. 

## 4. Treatment of Hyperacusis within the ASD Population

### 4.1. Habituation Training

Management of hyperacusis within the high functioning ASD population is relatively comparable to neurotypical patients. Treatment approaches often include application of sound therapy in combination with custom soundtracks or sound generating devices and extensive habituation training [50]. Such approaches are implemented to retrain one’s brain, specifically, the emotional and non-classical auditory pathway in order to reduce fearful responses to sound. In addition, it is important to desensitize an autistic child with hyperacusis to sounds by reducing use of unnecessary ear protection, as use of protection only helps to reduce symptoms of hyperacusis rather than tackling the cause of the sensitivity [50,51]. However, this desensitization should be done with more tact and in a more gradual timeline. Autistic children may need initially to have the option to protect themselves against the hyperacusis with noise cancelling headphones, and later on working closely with their parent, clinicians can start to implement desensitization. In order to directly approach the problem and desensitize to sound, the typical approach is to start by enriching the auditory system with pleasant sounds, then begin desensitization training to the undesirable sounds [50]. This is done by compiling a list of sounds that are bothersome to the patient, downloading the sounds to a music player, and instructing the patient to listen to the sounds at a low level, slowly increasing the volume each week. According to Danesh and colleagues, this approach is effective in reducing negative reactions to sounds in the autistic population [50].

### 4.2. Cognitive Behavioral Therapy

Cognitive behavioral therapy (CBT) for hyperacusis teaches the patient the skills and knowledge required to modify their noise-related irrational thoughts and safety seeking behaviors [52,53]. CBT is largely individualized, most often taking a client-centered approach. 

Juris, Andersson, Larsen, and Ekselius [54] analyzed the efficacy of psychologist-delivered CBT for 60 participants who reported hyperacusis as their primary complaint. The study was a randomized control trial design, with the experimental group receiving six weeks of CBT and the control group being placed on a wait list to receive therapy after six months. Participants were assessed using Loudness Discomfort Level, Hyperacusis Questionnaire [14] the Hospital Anxiety and Depression Scale [55], the Quality of Life Inventory [56] and an adapted version of the Tampa Scale of Kinesiophobia [57]. Results demonstrated significant between-group effect sizes which supported cognitive behavioral therapy, and follow up after 12 months showed that improvements were maintained [54]. 

An audiologist-delivered CBT approach for hyperacusis was introduced by Aazh and Allott in 2016 [52]. This approach utilized tasks including the following: discussion of emotional distress attributed to hyperacusis, creation of a behavioral experiment between clinician and patient to address irrational thoughts and fears, review of the outcome of the experiment, completion of a diary of thoughts and feelings, and working toward acceptance of emotional and physical symptoms related to hyperacusis [52]. 

Aazh and Moore (2018) determined the efficacy of CBT delivered by audiologists for 68 patients with tinnitus and/or hyperacusis in the United Kingdom. This study was retrospective in design, utilizing a service evaluation survey on all patients seen at the Tinnitus and Hyperacusis Therapy Specialist Clinic in 2015 [53]. Participants were assessed using pure tone audiometry (PTA), uncomfortable loudness level (ULL) testing, and questionnaires including the Tinnitus Handicap Inventory (THI) [22], Hyperacusis Questionnaire (HQ) [14], Insomnia Severity Index (ISI) [58], and Visual Analog Scale (VAS) [59]. Participants received six sessions of audiologist-delivered CBT focusing on discussion of patients’ reactions to their hyperacusis and developing behavioral enhancement strategies. Results demonstrated statistically significant improvements on all measures, with corrected effect size values ranging between 0.76 and 1.13 [53]. Further, Aazh, Bryant and Moore [60] evaluated the views of 31 patients who completed audiologist-delivered CBT for hyperacusis and/or tinnitus at a tinnitus and hyperacusis clinic over a one-year period. These patients received a survey questionnaire upon completion of their final session in order to provide feedback on their treatment. It was found that the majority of patients in this group felt CBT was effective, with a median response of 8/10, and that patients were able to manage their tinnitus/hyperacusis well, with a median response of 9/10 [60]. This study demonstrates efficacy of CBT for decreased sound tolerance disorders from the patient perspective.

With regard to the ASD population, there are no current studies demonstrating the efficacy of CBT for hyperacusis with regard to autistic people specifically. However, Storch and colleagues (2015) implemented a randomized control trial assessing the efficacy of CBT as a treatment approach for autistic early adolescents with concomitant anxiety [61]. Participants included 31 autistic adolescents aged 11 through 16 with a diagnosis of clinically significant anxiety. Participants were randomly assigned to the experimental group, which received 16 weeks of CBT, or the treatment as usual group, where participants received their typical treatment protocol. Treatment sessions involved the development of coping skills and eight sessions of exposure therapy. Results of this study revealed statistically significantly greater improvement in anxiety levels in the CBT group as compared to the treatment as usual group [61]. These results were maintained at one month follow up. This study suggests that autistic individuals respond well to cognitive behavioral therapy aimed at treating anxiety, which is a major component of hyperacusis treatment. It is important to know that the CBT intervention will need adjustments and modifications when clinicians face learning difficulties.

### 4.3. Auditory Integration Training

A more widely known and controversial treatment approach is known as Auditory Integration Training (AIT). This approach was, for a time, very popular among parents of autistic children [62,63,64]. There are conflicting findings in the literature regarding this approach, rendering it controversial and not supported by some professional organizations [62]. This treatment process begins by assessing for hyperacusis using audiometric testing to determine if the subject has “hypersensitive hearing” or “uneven hearing.” Upon determining eligibility, patients begin receiving treatment, which consists of 20 thirty-minute sessions conducted twice per day for 10 to 12 days [62]. Sessions consist of listening to music that filters out the frequencies that are determined to be sensitive as measured by peaks in the audiogram [63]. Progress is measured by changes in the audiogram and changes in behavior. As patients progress, these filters are modified.

Although previous literature reports improvements [63,65], no randomized control trials have supported its efficacy. A pilot study was conducted in to establish the efficacy of this management approach [16]. This study administered the AIT protocol for 17 autistic children and young adults aged between 4 and 21. The participants all had a primary diagnosis of autism from and independent agency. Assessment of hyperacusis consisted of parent ratings on the Hyperacusis Sensitivity Questionnaire, a tool developed by the researchers. The participants were split into experimental and placebo groups, with the experimental group receiving AIT and the control group listening to music that was not modified to filter out frequencies. Results suggested that the AIT group improved significantly in terms of repetitive behaviors, irritability, hyperactivity, and attention [28]. However, it is important to note that these two groups were not described in terms of comparability and were not randomly assigned to each group; in fact, the AIT group had statistically significantly different baseline data before beginning treatment. Results also demonstrated no improvement in hyperacusis as measured by their home developed questionnaire and use of the Pure Tone Discomfort (PTD) test [16]. Overall, this study did not demonstrate efficacy of the AIT approach.

To expand upon the aforementioned study, Rimland and Edelson conducted a larger scale study including 445 autistic children and adults as their primary or secondary diagnosis [66]. Participants were assessed using pure tone audiometry, and it was found that only 45% of participants were able to provide reliable responses. Frequency Discomfort testing was implemented by allowing a rater to score verbal and nonverbal reactions to sounds in order to provide a score. Participants were claimed to be randomly assigned to different experimental groups using different AIT devices, however, later in the article it was noted that participants were placed based on their assessment performance. Results of this study demonstrated a significant improvement of sound sensitivity as measured by the Frequency Discomfort testing and suggested that AIT improved the participants’ hearing by less than 1 decibel [66]. These results are biased by the subjective rating of frequency discomfort level, and it is important to note that 1 decibel is a clinically insignificant change. The fact that there was no control group also lessened the credibility of the results.

Zollweg, Palm, & Vance assessed the efficacy of AIT within a group of 30 autistic children and young adults [67]. Participants were placed in experimental or placebo groups similar to that of Rimalnd & Edelson’s pilot study [16]. There was no control group. This study assessed hyperacusis severity using behavior rating scales, pure tone audiometry, and Loudness Discomfort Level testing. Results of this study demonstrated no significant improvement of hyperacusis [67]. This study utilized a small sample size and did not use a control group; however, it exemplifies the conflicting findings of this treatment approach. Soon after this approach grew in popularity and results continued to draw controversy, the American Academy of Pediatrics published a statement which outlined the claims of efficacy and stated that further research is needed before this approach should be implemented [62].

## 5. Conclusions

In general, our knowledge about hyperacusis and other types of decreased sound tolerance disorders in autistic individuals continues to evolve as more research is conducted. Current research has shown that hyperacusis is highly prevalent in autistic people, adversely impacting social and academic domains due to sensory-based reactions to auditory stimuli. Research continues to grow and develop theories of the source of hyperacusis in autistic individuals, from genetics, to anatomy, to sensory processing capacities. It has been established that habituation training and cognitive behavioral therapy are effective treatment approach to treat hyperacusis. Conversely, as noted, auditory integration training has little credible data demonstrating its effectiveness. Increased awareness of hyperacusis, expansion of the body of literature, and improvements with research design over time have led to stronger evidence-based intervention approaches for this condition, and ongoing research is warranted to continue improving best practices. Further, additional research is necessary to assess hyperacusis treatment approaches specifically within the autistic population. In addition to ongoing research, application of newer genetic tests, neuroimaging techniques, and electrophysiological measurements may provide further insights into diagnosis and treatment of hyperacusis and other types of decreased sound tolerance disorders in this population. 

## Figures and Tables

**Table 1:** ASD Characteristics and Prevalence of Hyperacusis.

	Participants	Age (Years)	Severity of ASD	Tool to Assess Hyperacusis	Hyperacusis%
**Rimland & Edelson (1995)** [16]	17 (11 males) (6 females)	4–21	Unspecified	Hearing Sensitivity Questionnaire was given to parents	Mild—53% Moderate—24% Strong—18%
**Rosenhall et al., (1999)** [18]	199 (153 males) (46 females)	Children Adolescents	Autism	Unspecified	18%
**Demopoulos & Lewine (2016)** [17]	60 (48 males) (12 females)	5–18	High-low functioning	Unspecified	37%
**Danesh et al., (2015)** [19]	55 (46 males) (9 females)	4–42	High-functioning (Asperger)	Hyperacusis Questionnaire	69%
